# Evaluating the impact of targeting livestock for the prevention of human and animal trypanosomiasis, at village level, in districts newly affected with *T. b. rhodesiense* in Uganda

**DOI:** 10.1186/s40249-016-0224-8

**Published:** 2017-02-06

**Authors:** Louise Hamill, Kim Picozzi, Jenna Fyfe, Beatrix von Wissmann, Sally Wastling, Nicola Wardrop, Richard Selby, Christine Amongi Acup, Kevin L. Bardosh, Dennis Muhanguzi, John D. Kabasa, Charles Waiswa, Susan C. Welburn

**Affiliations:** 1 Edinburgh Infectious Diseases, Division of Infection and Pathway Medicine, Edinburgh Medical School: Biomedical Sciences, The University of Edinburgh, Chancellor’s Building, 49 Little France Crescent, Edinburgh, EH16 4SB UK; 2 Department of Pharmacy, Clinical and Comparative Medicine, School of Veterinary Medicine and Animal Resources, Makerere University, P.O. Box 7062, Kampala, Uganda; 3 The Coordinating Office for Control of Trypanosomiasis in Uganda (COCTU), Wandegeya, Plot 76/78 Buganda Road, P.O. Box 16345, Kampala, Uganda

**Keywords:** African animal trypanosomiasis (AAT), Human African trypanosomiasis (HAT), Sleeping sickness, *Trypanosma brucei rhodesiense* HAT (rHAT), Uganda, *T. b. brucei*, *T. b. rhodesiense*

## Abstract

**Background:**

Uganda has suffered from a series of epidemics of Human African Trypanosomiasis (HAT), a tsetse transmitted disease, also known as sleeping sickness. The area affected by acute *Trypanosoma brucei rhodesiense* HAT (rHAT) has been expanding, driven by importation of infected cattle into regions previously free of the disease. These regions are also affected by African Animal Trypanosomiasis (AAT) demanding a strategy for integrated disease control.

**Methods:**

In 2008, the Public Private Partnership, Stamp Out Sleeping Sickness (SOS) administered a single dose of ﻿try﻿p﻿anocide﻿ to ﻿31 486﻿ head of cattle in 29 parishes in Dokolo and Kaberamaido districts. This study examines the impact of this intervention on the prevalence of rHAT and AAT trypanosomes in cattle from villages that had (HAT^+ve^) or had not (HAT^-ve^) experienced a recent case of rHAT. Cattle herds from 20 villages were sampled and screened by PCR, pre-intervention and 6-months post-intervention, for the presence or absence of: *Trypanosoma brucei* s.l.; human infective *T. b. rhodesiense*; *Trypanosoma vivax*; and *Trypanosoma congolense* savannah.

**Results:**

Post-intervention, there was a significant decrease in the prevalence of *T. brucei* s.l*.* and the human infective sub-species *T. b. rhodesiense* in village cattle across all 20 villages. The prevalence of *T. b. rhodesiense* was reduced from 2.4% to 0.74% (*P* < 0.0001), with the intervention showing greater impact in HAT^-ve^ villages. The number of villages containing cattle harbouring human infective parasites decreased from 15/20 to 8/20, with *T. b. rhodesiense* infection mainly persisting within cattle in HAT^+ve^ villages (six/eight). The proportion of *T. brucei* s.l. infections identified as human infective *T. b. rhodesiense* decreased after the intervention from 8.3% (95% *CI* = 11.1–5.9%) to 4.1% (95% *CI* = 6.8–2.3%). Villages that had experienced a recent human case (HAT^+ve^ villages) showed a significantly higher prevalence for AAT both pre- and post-intervention. For AAT the prevalence of *T. vivax* was significantly reduced from 5.9% to 0.05% post-intervention while the prevalence of *T. congolense* increased from 8.0% to 12.2%.

**Conclusions:**

The intervention resulted in a significant decrease in the prevalence of *T. brucei* s.l*.*, human infective *T. b. rhodesiense* and *T. vivax* infection in village cattle herds. The proportion of *T. brucei* s.l. that were human infective, decreased from 1:12 *T. brucei* s.l. infections before the intervention to 1:33 post-intervention. It is clearly more difficult to eliminate *T. b. rhodesiense* from cattle in villages that have experienced a human case. Evidence of elevated levels of AAT in livestock within village herds is a useful indicator of risk for rHAT in Uganda. Integrated veterinary and medical surveillance is key to successful control of zoonotic rHAT.

**Electronic supplementary material:**

The online version of this article (doi:10.1186/s40249-016-0224-8) contains supplementary material, which is available to authorized users.

## Multilingual abstracts

Please see Additional file 1 for translations of the abstract into the five official working languages of the United Nations.

## Background

Human African Trypanosomiasis (HAT) or sleeping sickness, comprises two distinct tsetse transmitted parasitic diseases, both of which are fatal if they are not treated: chronic, *Trypanosoma brucei gambiense* HAT (gHAT) and acute *Trypanosoma brucei rhodesiense* HAT (rHAT) [1]. Uganda has active foci for both rHAT and gHAT which have been geographically separated for more than a century [1, 2]. gHAT lies in the northwest in districts adjacent to South Sudan and is relatively stable [3] while the area affected by rHAT, originally restricted to the shores of Lake Victoria [4] began expanding from the mid-1980s. An rHAT epidemic began in Tororo district in the mid-1980s spreading to Butaleja and Busia districts [5–7] spreading to Soroti and Serere districts in 2000 [7, 8] and subsequently to Kaberamaido and Dokolo districts by 2004 [9, 10]. By 2005, the areas affected by gHAT and rHAT were only 150 km apart [11].

The causal agent of rHAT, *T. b. rhodesiense,* co-exists in a range of non-human hosts [12] with a suite of other trypanosomes that cause African Animal Trypanosomiasis (AAT). A series of studies showed that cattle in Uganda carried human infective *T. b. rhodesiense* [6, 7, 9] and that movements of infected cattle from districts within the rHAT focus to rHAT free districts [8, 13] were responsible for the spread of rHAT in Uganda. The epidemic in Soroti was directly linked to importation of cattle through Brooks Corner cattle market [8].

Both HAT and AAT are major priorities for Uganda [14, 15] with approximately one-third of the national herd at risk from AAT [15], with serious economic losses [16]. Infection with *T. b. rhodesiense* in indigenous cattle is largely asymptomatic; livestock in rural areas of Uganda are not routinely treated unless they show co-infection with *T. congolense* or *T. vivax*. Cattle are long-term investments and untreated infected animals comprise a persistent zoonotic reservoir of rHAT parasites posing a threat to human health.

**Fig. 1:**
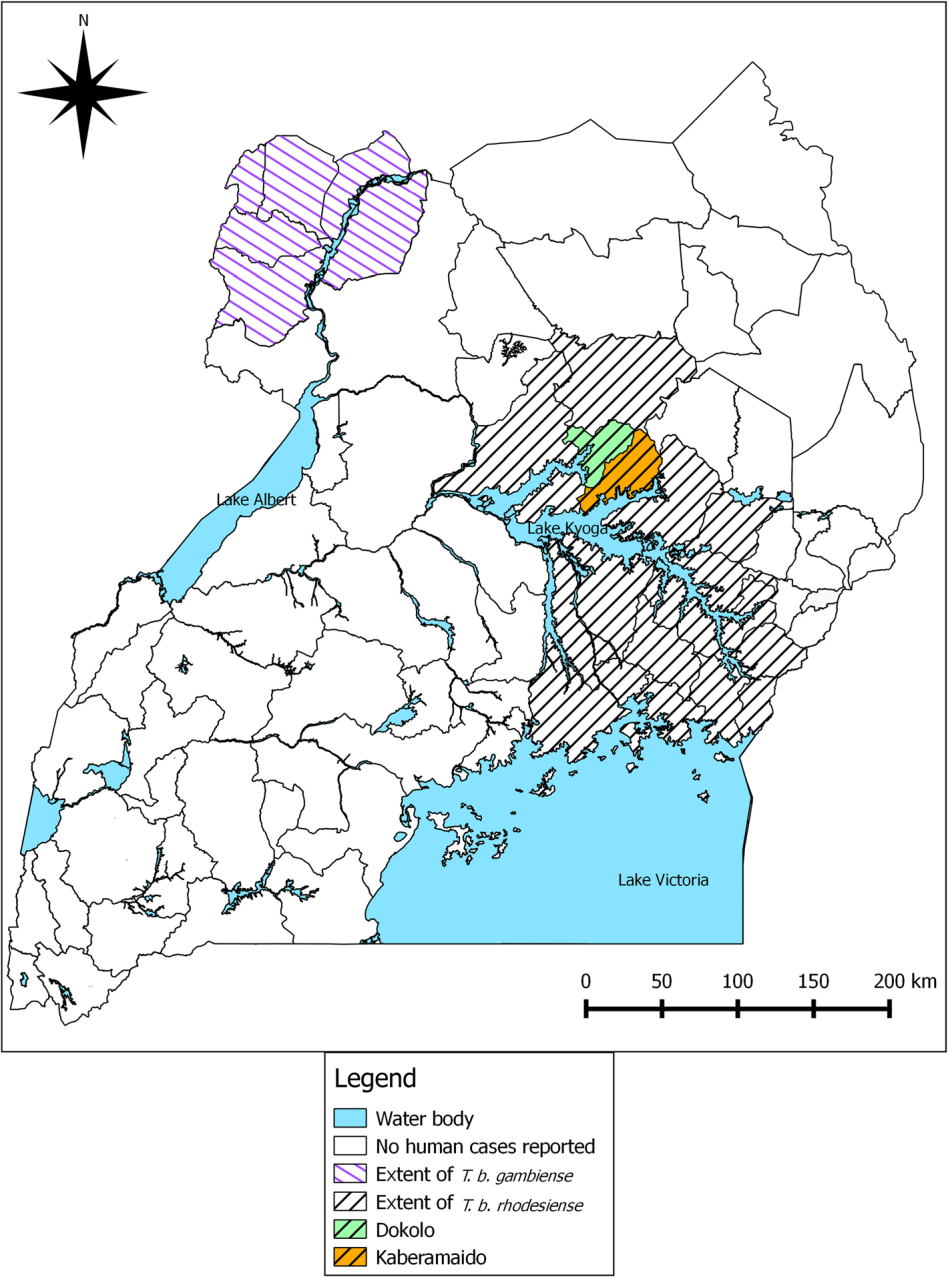
Districts of Uganda affected by *T. b. rhodesiense* HAT (rHAT) and *T. b. gambiense* HAT (gHAT). Study districts, Dokolo and Kaberamaido, are indicated

The expansion of the rHAT focus poses a significant health risk (Fig. 1) and convergence of the gHAT and rHAT foci would compromise existing approaches for diagnosis and treatment [17]. *T. b. rhodesiense* HAT is significantly under-reported [18] and identification of human cases remains a challenge to the health system [19]. Controlling rHAT requires veterinary intervention [20]. A single trypanocidal intervention, targeted to cattle in Kamuli and Soroti d﻿istricts, at high risk for rHAT in 2002 reduced the prevalence of *T. b. brucei* s.l. and *T. b. rhodesiense* and resulted in a significant decrease in human rHAT cases [21] suggesting that if sufficient head of cattle were treated, rHAT transmission could be stopped [22]. In 2006, a Public Private Partnership, Stamp Out Sleeping Sickness (SOS) [23], aimed to remove the reservoir of *T. b. rhodesiense* in Soroti, Dokolo, Lira, Apac and Kaberamaido districts by mass treating cattle [24].

Here we examine the impact of mass cattle trypanocidal intervention on the parasite burden (*T. brucei* s.l.*, T. vivax* and *T. congolense* and human infective *T. b. rhodesiense* parasites) in cattle within the SOS intervention zone, at village level, examining villages that had and had not previously experienced a human case of rHAT.

## Methods

### Description of the study area

The study area comprised Kaberamaido and Dokolo districts (in the Eastern and Northern regions respectively) in Uganda, which have been affected by rHAT since 2004. The districts border the northern shore of Lake Kyoga with a combined area of approximately 2 467 km^2^ and a human population of approximately 261 000 [25]. The main economic activities within the study area are agriculture and fishing, with the majority of the population engaged in subsistence farming [26]. The first cases of rHAT were reported in Kaberamaido and Dokolo districts in 2004 and the continued presentation of new cases indicates active transmission in both districts. A mass chemotherapuetic intervention was ﻿unde﻿rta﻿ken﻿ in cattle,﻿ ﻿in these districts in 2006, in an attempt to prevent the northwards spread of rHAT [24]. According to the livestock census undertaken in 2008, Dokolo had 58 902 cattle and Kaberamaido 76 109 cattle (Table 1).

**Table 1:** Demographic data for Dokolo and Kaberamaido districts

District	Human population (2002 census)	Cattle population (2008 census)	Land area (km^2^) (1995 census)	Estimated cattle density (km^2^)
Dokolo	129 385	58 902	1 113	53
Kaberamaido	131 650	76 109	1 354	56

N.B. Figures derived from the Ugandan Bureau of Statistics. Land mass for Kaberamaido excludes open water areas (269 km^2^ for) but includes seasonal and permanent wetlands (144 km^2^). Land/water area data for Dokolo district was not available, but this district has relatively small open water and wetland areas

### Description of the intervention

Between April and May 2008, due to the persistence of rHAT cases in p﻿arishes﻿ ﻿lying﻿ ﻿on the border between D﻿okolo﻿ and Kabaramiado [10, 27], a further intervention, comprising a single dose of trypanocide, was given to 31 486 cattle across 29 rHAT affected parishes (Table 2). All villages in the districts were geo-referenced using hand held global positioning systems prior to the intervention (GPS: Garmin, E-trex venture) [26]. Intervention sites were selected at parish level (between 3-10 villages); an average village had less than 100 households with between 30 and 50% of households owning cattle, typically small herds of 3–10 cattle although some larger herds of 50–100 cattle were present [28]. All available village cattle were injected with a single dose of Veriben (diminazene aceturate - Ceva Santé Animale) at 7.5 mg/kg; at the same time a single application of the insecticide Vectocid (Ceva Santé Animale) was applied to the legs and belly [29–31]. Treatment coverage data was obtained from estimates based on the total number of cattle treated in the parishes under the SOS intervention and from farmer reporting of intervention status of cattle sampled 6 months after intervention.

**Table 2:** Cattle treated March to April 2008 in Dokolo and Kaberamaido districts (percentage of the estimated total cattle population by parish)

Dokolo district				Kaberamaido district			
Parish	Estimated population	Number treated	%	Parish	Estimated population	Number treated	%
Anwangi/Amwoma	2 503	1 962	78	Oguolo	1 206	1 998	166
Aneralibi	1 252	1 044	83	Amoru	840	3 036	361
Iguli	1 050	867	83	Kalaki	1 554	903	58
Adwila	1 638	660	40	Kadie	752	1 554	207
Angwenya	400	1 253	313	Opiltok	162	1 701	1 050
Akurolango	1 242	2 117	171	Lwala	561	909	162
Angwecibange/Atur	882	656	75	Olelai	1 311	1 538	117
Alwithmac	800	760	95	Oryamo	584	1 261	216
Adagmon	1 000	488	49	Abalang	1 500	701	47
Awiri	513	578	127	Acanpi	790	420	53
Aderolongo	529	435	82	Kamuk	1 503	783	52
				Okile	972	2 466	254
				Abal-kweru	1 037	993	96
				Kaberamaido	873	188	22
				Plantau	1 804	1 051	58
				Katinge	1 470	1 164	79
TOTAL	11 809	10 820	91.6%	TOTAL	16 919	20 666	122%

### Study design

**Fig. 2:**
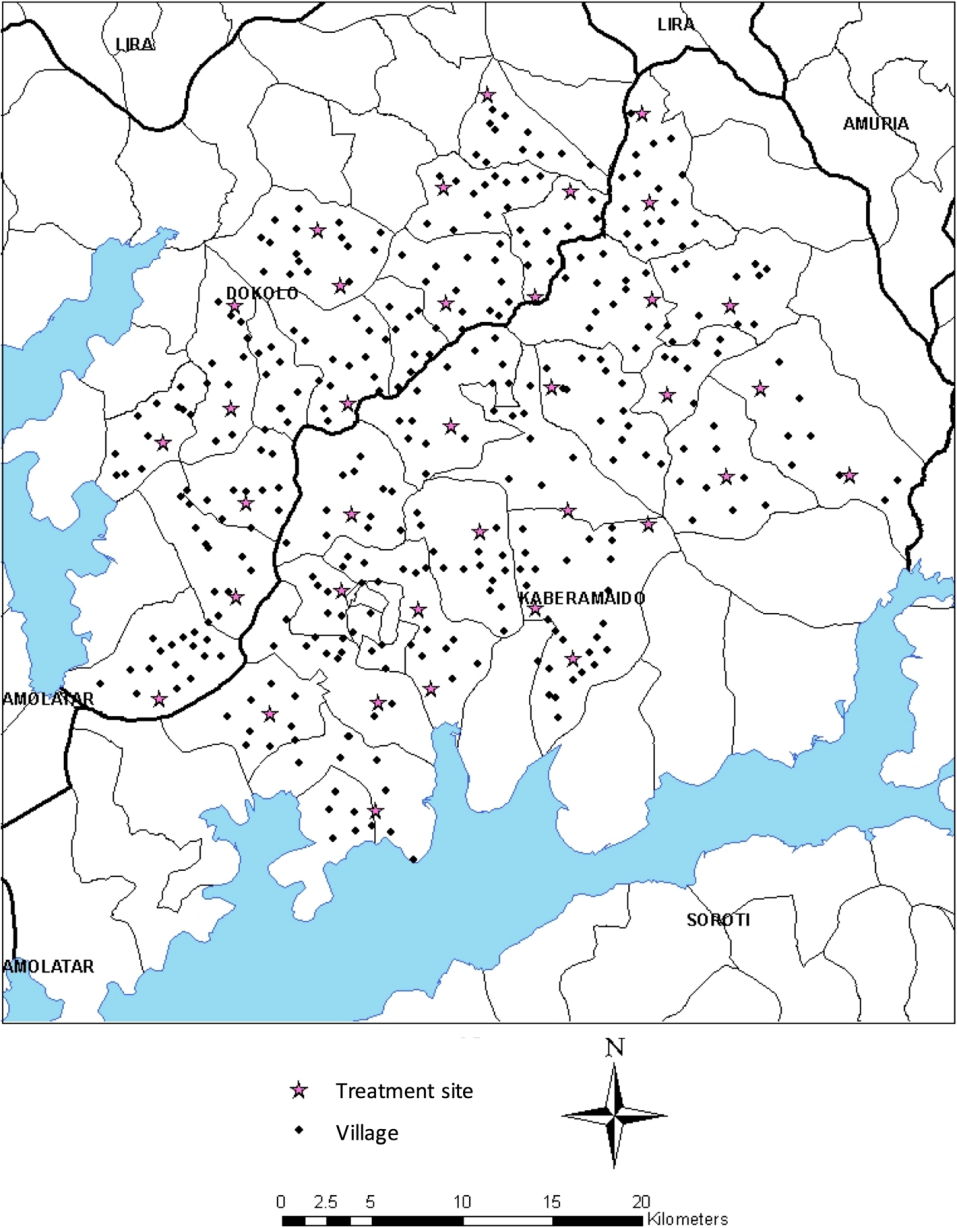
Location of study village and intervention (treatment sites) in Dokolo and Kaberamaido districts

**Fig. 3:**
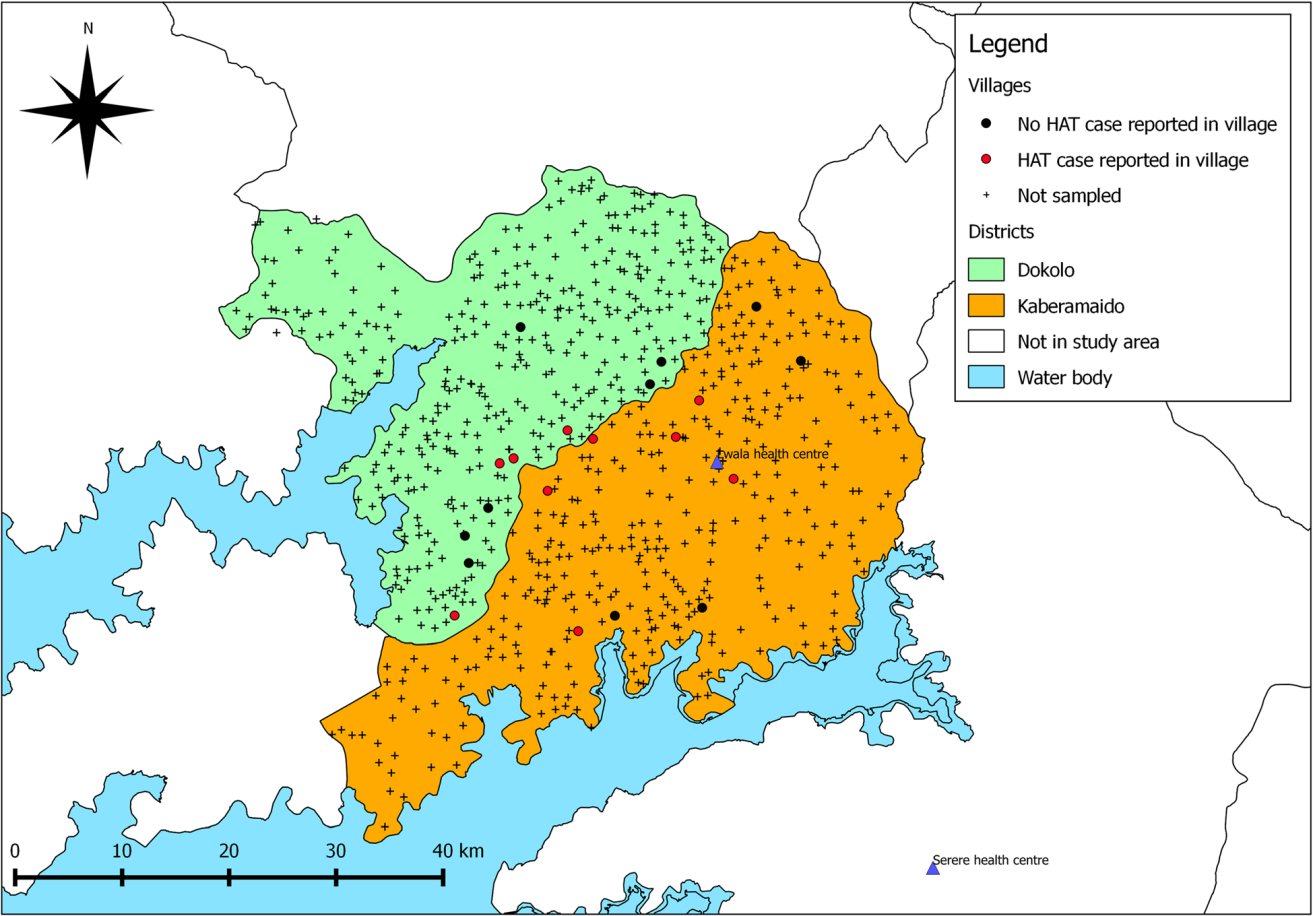
Location of HAT^+ve^ and HAT ^-ve^ study villages in Dokolo and Kaberamaido districts

To examine the parasite dynamics before and after intervention, two groups of villages were selected: villages that had experienced a case of human rHAT in the previous 6 months (rHAT^+ve^ villages) and those that had never reported a case (rHAT^-ve^ villages). Records detailing all rHAT patients between January and June 2007 were obtained from the two health centres established for treatment of rHAT in Dokolo and Kaberamaido: Lwala health centre in Kaberamaido district and Serere health centres in Soroti district [18]. Cases were cross-referenced to the geo-referenced village data to classify all villages into two groups: case villages and no-case villages. A case village (rHAT^+ve^ village) was defined as having one or more rHAT cases diagnosed in the 6 month period January – June 2007. Twenty villages were randomly selected to monitor the impact of the intervention: 10 rHAT^+ve^ villages that had reported a rHAT case between January – June 2007 and 10 that had never reported a case (HAT^-ve^ villages). Figure 2 shows the location of all study villages and the intervention sites. The village HAT case status is shown in Fig. 3 which also indicates the location of the HAT treatment centre.

### Sample size calculation

The required sample size was based on the requirement for comparing two proportions (rHAT case vs. non rHAT case villages) [32], with adjustment for cluster sampling [33] at the village level. An estimated *T. brucei* s.l. prevalence of 20% was used, alongside an inter-cluster variance (V_c_) of 0.013 [34] to allow for a differences in trypanosome prevalence to be detected at 95% confidence and 80% power [32]. This called for a minimum sample number of 67 individual cattle per cluster, across 20 village clusters (1 340). Cattle were systematically sampled by order of presentation at each village sampling site, up to a maximum of 100 animals per village, to maintain sufficient sample size of cattle from rHAT case and non-rHAT case villages overall, and to compensate for villages with fewer than 67 cattle.

### Sample collection

Samples of blood (100 μl) were taken from the ear vein of cattle in the rHAT+ve (case) villages and rHAT+ve (no case) villages, before (April – May 2008) and 6 months after intervention (October 2008). In total 3 549 cattle were sampled: 1 658 before intervention and 1 891 after 6 months intervention. Before taking blood samples, the livestock owner was asked whether the animal had been treated during the mass intervention.

One hundred microliters of blood was drawn from the ear capillary using a lancet, collected into two 50 μl capillary tubes and spotted on a FTA™ card (Whatman, Maidstone, Kent, UK). Cards were air dried at room temperature for 24 h and stored in multi-barrier pouches with desiccant (Whatman, UK).

### Laboratory analysis

Samples were analysed by PCR to identify all trypanosome species and subspecies present. Five 3 mm discs were punched from each blood sample and processed as previously described [35–37]. Discs were washed four times, twice with FTA purification reagent and twice with TE buffer, before being air dried and then heated at 90 °C for 30 min suspended in 5% (w/v) Chelex solution. Five microliters of eluted sample solution, was used to seed all PCR reactions. Previously published species specific primers were used to detect *T. brucei* s.l., *T. vivax*, and *T. congolense* savannah, in three separate PCR reactions [35–37]. Samples positive for *T. brucei* s.l. were further tested for *T. b. rhodesiense* using a multiplex PCR to discriminate *T. b. brucei* and *T. b. rhodesiense* [38]. Standard PCR amplifications for all reactions were carried out in 25 μl mixtures; reaction conditions, primer sequences and cycling conditions were as previously published [35–37]. One positive control [genomic deoxyribonucleic acid (DNA)] and one negative control (extract from blank FTA™ disc) were run with each reaction. PCR products were resolved via electrophoresis on 1.5% agarose gels using GelRed DNA stain and run at 100v for a minimum of 45 min in the presence of a molecular marker, until band size could be easily determined. PCR products were visualised and documented with a BioRad GelDock™ imaging system.

### Statistical analysis

The prevalence of *T. brucei* s.l., *T. b. rhodesiense, T. vivax* and *T. congolense* savannah trypanosome infection detected by PCR were expressed as a percentage, and exact binomial 95% confidence intervals were computed (R, version 2.0.1). Fisher’s Exact test was used to compare trypanosome prevalence in rHAT^+ve^ and rHAT^–ve^ villages and between th﻿e pre- and 6 months post-intervention sampling.

## Results

One thousand six hundred and fifty-eight cattle blood samples were obtained before intervention from the 20 village sites; 854 from animals in HAT^+ve^ villages and 804 from cattle in HAT^–ve^ villages. Of these, 642 samples were positive for one or more species of trypanosome; 362 from HAT^+ve^ villages (prevalence 42.4%, 95% *CI* = 45.8–39.1%) and 280 were from HAT^–ve^ villages (prevalence 34.8%, 95% *CI* = 38.2–31.5%). The prevalence of cattle trypanosomiasis (AAT) was significantly higher in HAT^+ve^ villages (*P* < 0.001).

**Fig. 4:**
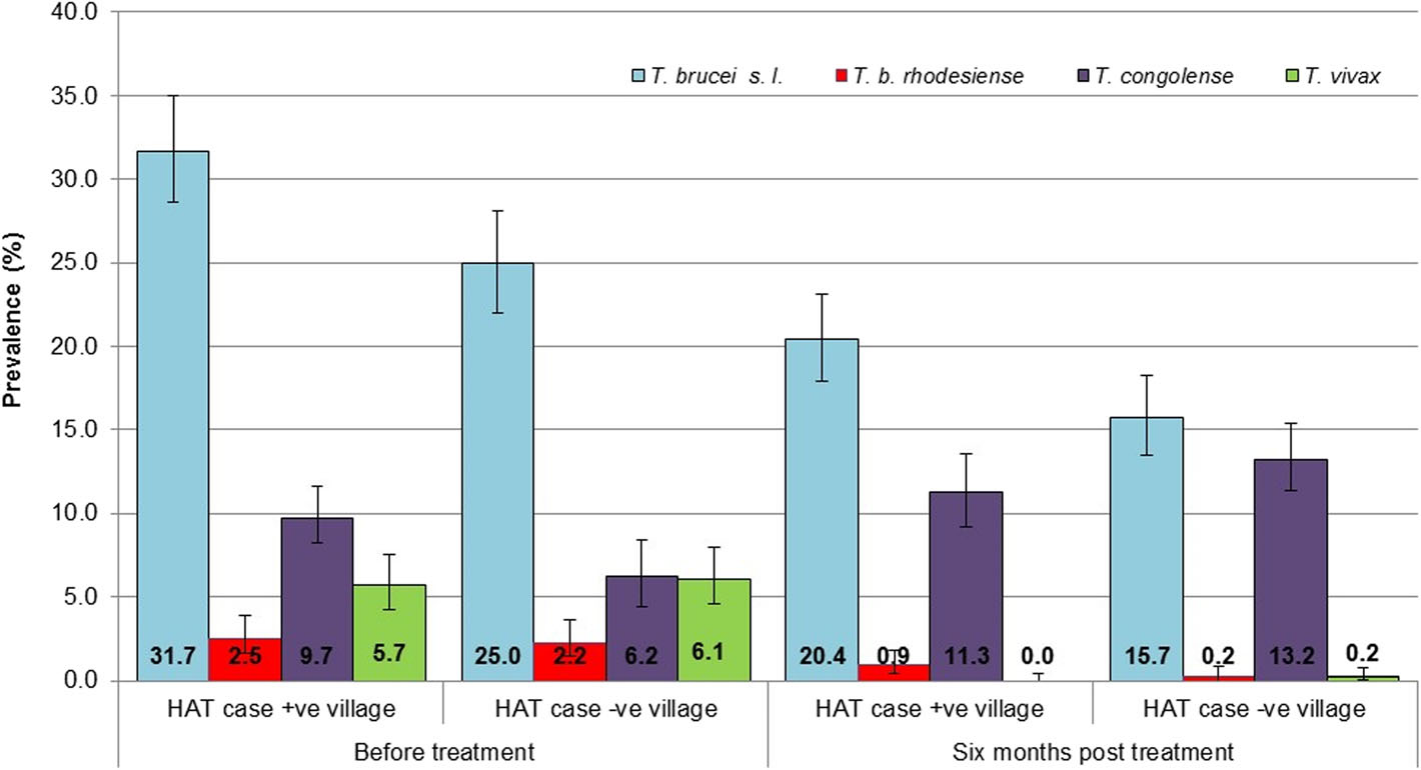
Prevalence ﻿o﻿f trypanosome species and sub-species (*T. brucei* s.l., *T. b. rhodesiense*, *T. congolense* and *T. vivax*) pre- and 6-months post-intervention

Six months post-intervention, 1 891 cattle blood samples were taken from the same 20 villages; 956 from HAT^+ve^ villages and 935 from HAT^–ve^ villages. Of these 509 cattle were positive for one or more species of trypanosome; 266 (prevalence 27.8%, 95% *CI* = 30.8–25.0%) were from HAT^+ve^ villages and 243 (prevalence 26.0%, 95% *CI* = 28.9–23.2%) from HAT^–ve^ villages (see Fig. 4).

### *T. brucei* s. l

*T. brucei* s.l. was the most common species detected both before and after intervention (Fig. 4). *T. brucei* s.l. prevalence varied from 15.6 to 53.0% between villages. The prevalence of *T. brucei* s.l. was significantly higher in HAT^+ve^ villages than in HAT^–ve^ villages pre-intervention (*P* = 0.002) and post-intervention (*P* = 0.008).

Across all 20 villages, the prevalence of *T. brucei* s.l. was significantly lower 6 months after the intervention, irrespective of village HAT status. For HAT^+ve^ villages, the prevalence of *T. brucei* s.l. in cattle was 31.7% (271/854 95% *CI* = 35.0–28.6%) pre-intervention and significantly lower, 20.4% (195/956 95% *CI* = 23.1–17.9%), post-intervention (*P* < 0.0001). In cattle from villages defined as HAT^–ve^, *T. brucei* s.l. prevalence was 25.0% (201/804 95% *CI* = 28.1–22.0%) pre-intervention and significantly lower, 15.7% (147/935 95% *CI* = 18.2–13.4%), 6 months post-intervention (*P* < 0.0001).

### *T. b. rhodesiense*

There was a reduction in the prevalence of human infective *T. b. rhodesiense* in cattle 6 months post intervention in both HAT^+ve^ and HAT^–ve^ villages. Across the 20 study villages the prevalence of human infective *T. b. rhodesiense* in cattle was reduced 3-fold, from 2.4% (39/1658) pre-intervention to 0.74% 6 months post-intervention (14/1891). Within the HAT^+ve^ villages, pre-intervention, the prevalence of *T. b. rhodesiense,* in cattle was 2.5% (21/854, 95% *CI* = 3.7–1.5%). This was halved to 1.26% (12/956, 95% *CI* = 2.2–0.7%, *P* = 0.077) post-intervention. In cattle from the HAT^–ve^ villages the prevalence of *T. b. rhodesiense* was 2.2% (18/804, 95% *CI* = 3.5–1.3%) pre-intervention, and this was significantly decreased, by more than 10-fold, post-intervention to 0.2% (2/935, 95% *CI* = 0.8–0.0%, *P* < 0.0001).

**Fig. 5:**
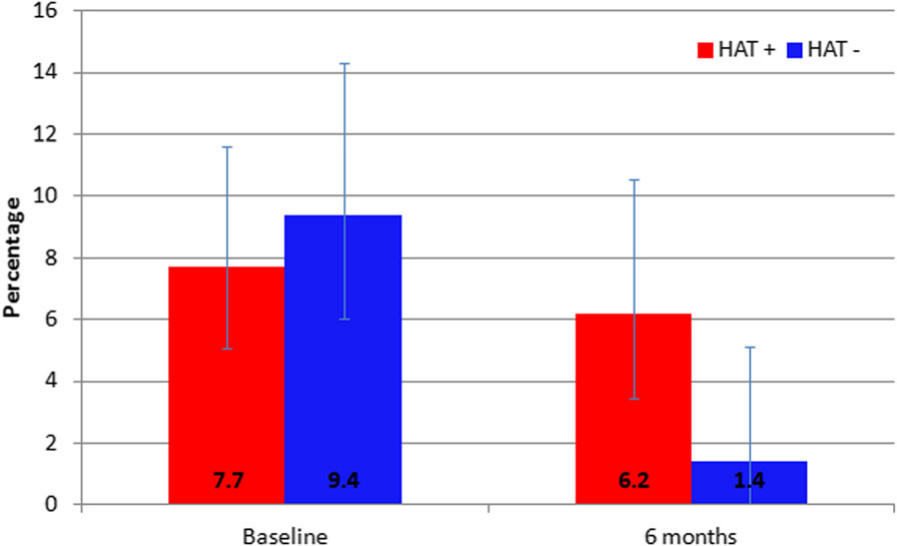
Proportion of *T. brucei* s.l. that are *T. b. rhodesiense* (infective for humans) pre- and post-intervention in HAT^+ve^ and HAT^-ve^ study villages (95% confidence intervals indicated)

**Fig. 6:**
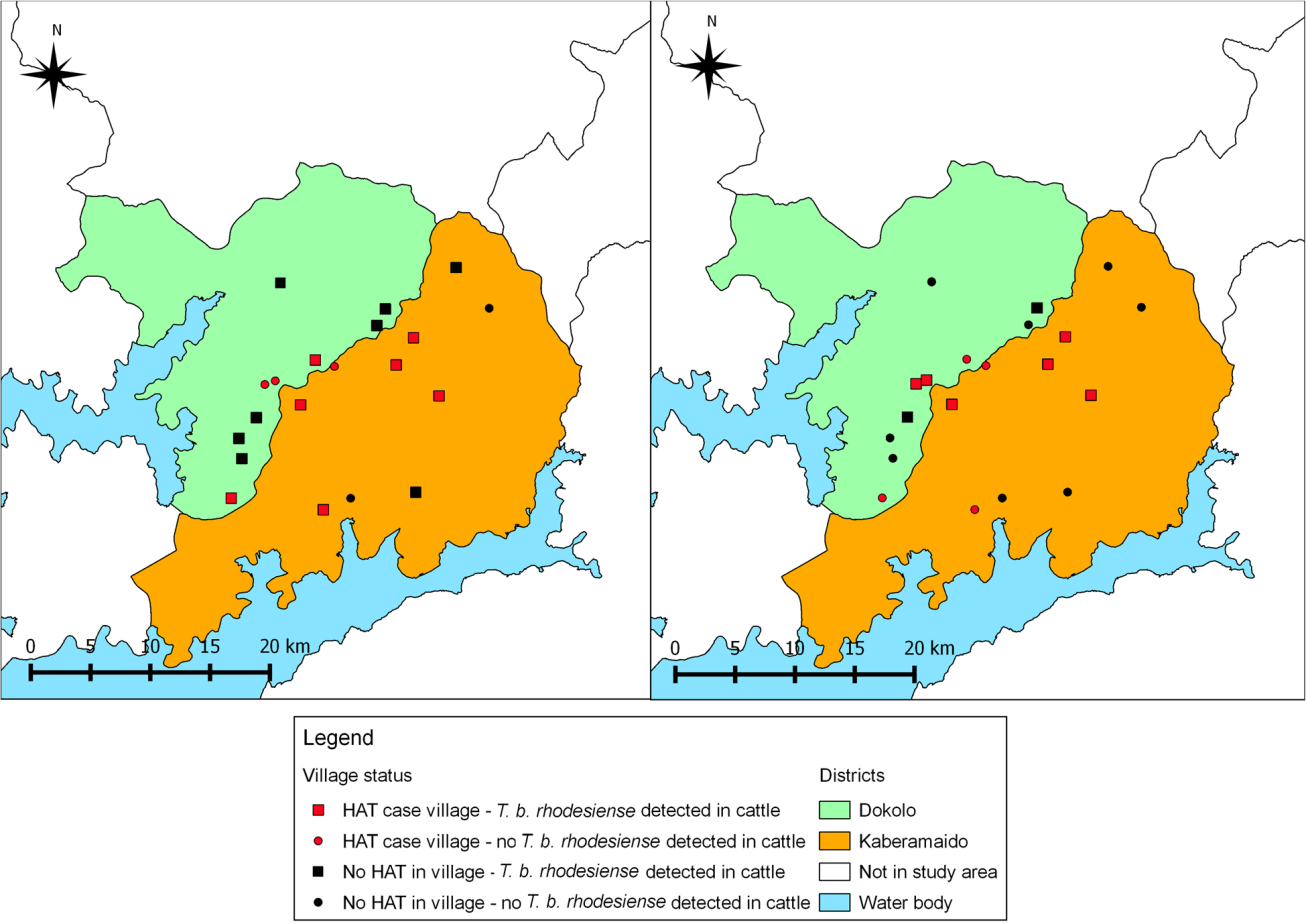
Distribution of villages harbouring *T. b. rhodesiense* infected cattle pre- and 6-months post-intervention

The intervention also impacted in the proportion of human infective *T. b. rhodesiense* within the population of circulating *T. brucei* s.l. in cattle (Fig. 5). Critically, the proportion of *T. b. brucei* that was *T. b. rhodesiense* (human infective) decreased after intervention. Prior to the intervention in rHAT^+ve^ and rHAT^–ve^ villages combined 8.3% (39/472, 95% *CI* = 11.1–5.9%) of circulating *T. brucei* s.l. were human infective; post-intervention this reduced to 4.1%, (14/342, 95% *CI* = 6.8–2.3%). Prior to the intervention, cattle in 15 of the 20 study villages had *T. b. rhodesiense* prevalences ranging from 1 to 5%. *T. b. rhodesiense* was not detected in any cattle in three HAT^+ve^ villages and two HAT^–ve^ villages. Six months post-intervention, only eight of the 20 study villages contained *T. b. rhodesiense* positive cattle; 75% (6/8) of these were from HAT^+ve^ villages. The locations of villages harbouring cattle infected with *T. b. rhodesiense* before and after intervention are shown in Fig. 6.

### AAT

Prior to the intervention, the prevalence of *T. vivax* in HAT^+ve^ village cattle was 5.7% (49/854, 95% *CI* = 7.5–4.3%) and 6.1% (49/804, 95% *CI* = 8.0–4.5%) in HAT^–ve^ villages. At 6 months post-intervention, no *T. vivax* infected cattle were found in the HAT^+ve^ villages (0/956, 95% *CI* = 0.4–0%, *P* < 0.0001) and only a single case of *T. vivax* in HAT^–ve^ villages was observed (0.1%; 1/935, 95% *CI* = 0.6–0.0%, *P* < 0.0001). This is a significant reduction in prevalence, from 6% (98/1658) pre-intervention to 0.05% (1/1891) 6 months after intervention. There was no significant difference in the prevalence of *T. vivax* in cattle from villages defined as HAT^+ve^ or HAT^–ve^ either before or after intervention.

Infection in cattle with *T. congolense* savannah was significantly greater than *T. vivax*, both before and after intervention (*P* = 0.0043) (Table 3). In total, the prevalence of *T. congolense* savannah increased from 8% (133/1 658) to 12.2% (231/1891). In cattle from HAT^+ve^ villages there was no significant difference in the prevalence of *T. congolense* savannah before (9.7%; 83/854, 95% *CI* = 11.9–7.8%) or after intervention (11.3%; 108/956, 95% *CI* = 13.5–9.4%) (*P* = 0.2842). In cattle from HAT^–ve^ villages the prevalence of *T. congolense* savannah before intervention was 6.7% (54/804, 95% *CI* = 8.7–5.1%), significantly lower than in HAT^+ve^ villages (*P* = 0.0319). Six months post-intervention, the prevalence of *T. congolense* savannah of 13.2% significantly increased (123/935, 95% *CI* = 15.5–11.1%, *P* < 0.0001).

**Table 3:** Prevalence of *Trypansoma vivax* and *Trypanosoma congolense* savannah species pathogenic for cattle, in HAT^+ve^ and HAT^–ve^ villages before and after intervention

	Village HAT status	Prevalence *T. vivax*	Prevalence *T. congolense* savannah
Pre-intervention	HAT^+ve^	5.7% (7.51–4.27%)	9.7% (11.91–7.82%)
	HAT^+ve^	6.1% (7.98–4.54%)	6.7% (8.67–5.09%)
	Total	5.9% (7.16–4.87%)	8.0% (9.43–6.81%)
Six months post-intervention	HAT^+ve^	0% (0.39–0%)	11.3% (13.48–9.36%)
	HAT^–ve^	0.1% (0.41–0.01%)	13.2% (15.48–11.13)
	Total	0.05% (0.30–0%)	12.2% (13.77–10.81%)

Brackets indicate 95% confidence intervals

### Intervention coverage

Mathematical modelling estimated that to interrupt *T. b. rhodesiense* transmission, where cattle were the principal reservoir of *T. b. rhodesiense*, required treatment of 86% of the cattle population [22]. Between April and May 2008, SOS reported administering a trypanocide to 31, 486 head of cattle across 29 parishes in Dokolo and Kaberamaido. Estimations of intervention coverage were based on the total number of cattle treated, the 2008 livestock census data and farmer based reporting 6 months post-intervention. The livestock census was based on household survey data collected at a fixed number of points within each district throughout Uganda; this was modelled to parish level, based on demographic trends and other data, by district-level staff. The exact methodology employed is uncertain, and the parish level data are of variable quality. The 2008 census data were conservatively inflated by 20% on the advice of the District Veterinary Officers in Dokolo and Kaberamaido to allow for population growth and importation from other districts as part of national restocking programs as well as trade (Table 2).

In Dokolo district, 10 820 out of an estimated 11, 809 head of cattle from 13 parishes were treated (91.6%) – 20% of the total estimated cattle population of Dokolo district (58 902). In Kaberamaido, 20 666 cattle were treated from 16 parishes with an estimated cattle population of 16 919 head, giving estimated coverage of 122% or 27% of the total cattle population in the district (76 109 head). Of the 29 parishes, eight showed coverage below 60% while 11 parishes showed coverage >100%; six parishes had more than 200% estimated coverage.

Six months after the intervention, livestock-keepers in the 20 study villages were asked to report whether their animals had participated in the intervention. According to the livestock-keepers, 55.2% (1 044/1891) of the cattle sampled 6 months after the intervention had participated in the intervention (Table 4). Fewer animals, 51% (486/956), were reported to have participated in the HAT^+ve^ villages than in the HAT^-ve^ villages, 59.7% (558/935).

**Table 4:** *Trypanosoma* infection and intervention participation status of cattle sampled at 6 months post-intervention as reported by livestock-keepers

Village HAT status	Intervention participation	Cattle sampled (*n*)	TBSL + (*n*)	% TBSL +	SRA + (*n*)	% SRA +	TV + (*n*)	% TV +	TC + (*n*)	% TC +
HAT + villages		956	195	20.4	12	1.3	0	0	108	11.3
	Unknown	32	5	15.6	0	0	0	0	3	9.4
	Intervention	486	89	18.3	4	0.8	0	0	56	11.5
	No intervention	438	101	23.1	8	1.8	0	0	49	11.2
HAT - villages		935	147	15.7	2	0.2	1	0.1	123	13.2
	Unknown	27	0	0	0	0	0	0	4	14.8
	Intervention	558	86	15.4	1	0.2	1	0.2	83	14.9
	No intervention	350	61	17.4	1	0.3	0	0	36	10.3

NB: TBSL + − sample positive for *T. brucei* s.l; SRA + sample positive for human infective *T. b. rhodesiense;* TV + sample positive for *T. vivax;* TC + sample positive for *T. congolense* savannah

Intervention coverage impacted on identification of *Trypanosoma* infection by PCR (Table 4). Of the 342 animals found positive for *T. brucei s.l.*, 47% at the 6 month post-intervention sampling were from cattle that had not participated in the intervention (162/342). Within the HAT^+ve^ villages 52% (101/195) of the animals found positive for *T. brucei,* had not participated in the intervention. Of the 12 *T. b. rhodesiense* infections identified in HAT^+ve^ villages, 66% (8/12) were observed in cattle reported not to have particpated in the intervention . Of the 231 *T. congolense* infections identified 37% were from animals reported not to have participated in the intervention and 63% from those reported to have particpat﻿ed.

## Discussion

Microscopy consistently underestimates the prevalence trypanosomes in livestock [38, 39], so the use of sensitive and specific ‘field friendly’ diagnostic tools are key to our understanding of the epidemiology of bovine and zoonotic trypanosomiasis [39]. Cattle have been shown to play a major role in the epidemiology of rHAT in Uganda [6–9, 21, 33]. This is one of the first studies to use molecular tools to evaluate large-scale field interventions targeted at *T. b. rhodesiense,* the causal agent of rHAT, in cattle - the principal animal reservoir. Previous studies have shown the importance of eliminating the reservoir of human infective *T. b. rhodesiense* parasites from domestic cattle to prevent human infection [21, 32].

Across the 20 randomly selected villages within the intervention zone we observed a significant reduction of *T. b. rhodesiense* in cattle 6 months after the intervention (from 2.40 to 0.74%), a 10-fold decrease in prevalence of *T. b. rhodesiense* in HAT^–ve^ villages compared to a 50% reduction of *T. b. rhodesiense* in HAT^+ve^ villages. The number of villages with *T. b. rhodesiense* infected cattle fell from 15 to eight villages post-treatment; most *T. b. rhodesiense* infected cattle post-intervention were from villages that had a case of human rHAT (six of the eight infected villages). This emphasises the focal nature of rHAT [33, 40] suggesting that once *T. b. rhodesiense* is established in a village, spilling over from cattle into humans, eliminating the cattle reservoir of *T. b. rhodesiense* in the village becomes more difficult.

HAT transmission is influenced by many factors, including tsetse population density, tsetse susceptibility [41] and proximity to around a central point such as a market, watering point or swampland [8, 10, 40]. In this study villages that had experienced a case of rHAT also showed a significantly higher prevalence of *T. brucei* s.l. and *T. congolense* in the village cattle than those that had not had a case of rHAT, further evidence of a link between AAT and *T. b. rhodesiense* HAT. Conditions for transmission of all species of trypanosome appeared more favourable in HAT^+ve^ villages.

*T. vivax* all but disappeared across the study villages post-intervention, but the prevalence of *T. congolense* increased (although a large proportion of infected animals had not participated in the intervention). Drug resistance in cattle has been reported for *T. congolense* in parts of Ethiopia [42] and Burkina Faso [43] but this has not been reported in Uganda where *T. congolense* infection is rarely reported or treated. Eliminating *T. vivax* and reducing *T. brucei* s.l. in cattle may simply offer opportunities for *T. congolense* to establish in these animals [44].

To interrupt *T. b. rhodesiense* transmission, where cattle are the principal reservoir of *T. b. rhodesiense*, it is calculated that 86% of the cattle population must be treated [22]. The discrepancy between the coverage estimates calculated from the livestock census data (91.3%) and the lower estimates obtained provided from the livestock-keepers (55.2%) show the difficulties of calculating coverage in the absence of robust and verifiable village and parish cattle numbers; true coverage probably lay somewhere between the two.

In Dokolo, cattle were treated in 13 of 33 parishes in the district, or 39% of the district; coverage was estimated at 20% of the district cattle. Likewise, for Kaberamaido, the mass intervention covered 50% of the district (16 of 32 parishes) but using, 2008 livestock census data, only 27% of the district cattle population are estimated to have been treated. The parishes treated during the mass intervention had relatively low cattle populations compared to other areas of the district.

Accurate information about the number of cattle owned by individuals and households in this region is difficult to obtain. Extensive cattle rustling in the late 1980s decimated livestock in the region and the Lango and Teso economy [45] and cattle restocking projects [46] were ongoing during 2008 when this intervention took place [13]. Villagers may answer questions about donor-led interventions in ways that take into account how their answers will influence future benefits [47] and it is likely that a proportion of livestock-keepers misrepresented the treatment status of their cattle in the hope that their herd would be re-treated.

## Conclusions

Treatment of cattle produced a significant decrease in the prevalence of *T. brucei* s.l*.*, human infective *T. b. rhodesiense* and *T. vivax* infection in village cattle herds and impacted on the proportion of *T. brucei* s.l. that were human infective: from 1:12 human infective *T. brucei* s.l. before the intervention to 1:33 post-intervention.

The observation that is more difficult to remove *T. b. rhodesiense* from villages that have experienced a rHAT case, makes a strong case for improved information sharing between human health providers, animal health providers and the community. The prevalence of AAT in villages can serve as an additional indicator of human health risk in these communities, alerting medical services to survey human populations. Incorporating participatory research in conjunction with traditional disease modelling approaches could lead to improved disease control [48].

To prevent the continued migration of rHAT, point of sale trypanocide treatments should be applied for all inter-district cattle movements and interventions that target rHAT and AAT should be scaled up in all rHAT affected districts. Interventions that additionally target tick-borne diseases are welcomed by local communities [45], where trypanosomiasis and tick-borne diseases reduce draft cattle output by 21% and household income from the use of oxen (estimated at $245 USD annually) by 32% [49]. Appropriate scaled application of trypanocidal drugs, followed by routine application of veterinary pyrethroids to prevent re-infection and manage tick infestation, offer a sustainable solution for zoonotic HAT, AAT and tick-borne disease control [29, 31, 49, 50] offering a win-win for human and animal health.

## Additional file

Additional file 1: Multilingual abstracts in the six official working languages of the United Nations. (PDF 862 kb)

## Abbreviations

AAT: Animal African Trypanosomiasis
COCTU: The Coordinating Office for Control of Trypanosomiasis in Uganda
DVO: District Veterinary Officer
gHAT: *T.b. gambiense* HAT
HAT: Human Animal Trypanosomaisis
ICONZ: Integrated Control of Neglected Zoonoses
LRA: Lord’s resistance army
PCR: Polymerase chain reaction
PPP: Public Private Partnership
rHAT: *T.b. rhodesiense* HAT
SOS: Stamp out Sleeping Sickness
