# Supplementary material for: Evaluating the impact of targeting livestock for the prevention of human and animal trypanosomiasis, at village level, in districts newly affected with T. b. rhodesiense in Uganda
Source: Infect Dis Poverty. 2017 Feb 6;6:16. doi: 10.1186/s40249-016-0224-8 (PMC5292814; doi:10.1186/s40249-016-0224-8)
Supplement: Additional file 1: — Multilingual abstracts in the six official working languages of the United Nations. (PDF 862 kb) [file 40249_2016_224_MOESM1_ESM.pdf]

Translation of the abstract into the five official working languages of the United Nations

تقييم أثر استهداف المواشي للوقاية من داء المثقبيات البشري والحيواني، على مستوى القرية، في المناطق المتضررة حديثاً بالسل الروديسي في أوغندا

لويز هاميل، كيم بيكوتزي، جينا فايف، بياتريس فون فيسمان، سالي ووسلينج، نيكولا وردروب، ريتشارد سيلبي، كريستين أمانجي أوكب، كيفن ل. باردوش، دينيس موهانجوزي، جون د. كاباسا، تشارلز ويسواند، سوزان سي. ويلبيرن

الملخص

الخلفية: تعانى أوغندا من سلسلة من الأوبئة بسبب داء المثقبيات الأفريقي البشري (HAT)، والمرض المنقول بذبابة التسي تسي ، المعروف أيضاً باسم مرض النوم. وتتوسع المنطقة المتأثرة بداء المثقبية البروسية الروديسية الحادة (HAT rHAT) ، المنقول باستيراد الماشية المصابة إلى مناطق كانت سابقاً خالية من المرض. وتتأثر هذه المناطق أيضاً بداء المثقبيات الأفريقي الحيواني (AAT) وتتطلب مكافحة متكاملة للأمراض.

المنهجية: في عام 2008، قامت شراكة القطاعين العام والخاص للقضاء على مرض النوم (SOS) بعلاج 31486 رأس ماشية بمضاد المثقبيات في 29 منطقة في مقاطعتي دوكولو وكابيرامايدو. وتتناول هذه الدراسة تأثير هذا التدخل على انتشار داء المثقبية البروسية الروديسية الحادة (rHAT) وداء المثقبيات الأفريقي الحيواني (AAT) في ماشية القرى التي شهدت (HAT^{+ve})) أو لم تشهد (HAT^{-ve}) حالة حديثة من داء المثقبية البروسية الروديسية الحادة rHAT. وتم أخذ عينات من قطعان الماشية من عشرين قرية وفحصها بواسطة تفاعل البوليميراز المتسلسل PCR، قبل التدخل و 6 أشهر بعد التدخل، لوجود أو عدم وجود: المثقبية البروسية. s.l، والسل الروديسي المعدي للإنسان، والمثقبية النشيطة، والمثقبية الكونغولية السافانا.

النتائج: بعد التدخل، كان هناك انخفاض كبير في انتشار المثقبية البروسية .s.l والأنواع الفرعية للسل الروديسي المعدي للإنسان في ماشية القرى عبر كل القرى العشرين. وانخفض انتشار السل الروديسي من 2.40٪ إلى 0.74٪ (P <0.0001)، وأظهر التدخل تأثيراً أكبر في قرى HAT^{-ve}. انخفض عدد القرى التي تحتوي على الماشية حاملة الطفيليات المعدية للإنسان من 20/15 إلى 20/8 مع استمرار عدوى السل الروديسي بصورة رئيسية في ماشية القرى HAT^{+ve} (8/6). وانخفضت نسبة عدوى المثقبية البروسية .s.l التي كانت بالتحديد السل الروديسي المعدي للإنسان بعد التدخل من 8.3٪ (95% فاصل ثقة= 11.1% - 5.9%) إلى 4.1٪ (95% فاصل ثقة= 6.8% - 2.3%). وأظهرت القرى التي شهدت حالة إصابة بشرية حديثة (قرى HAT^{+ve}) معدلات انتشار أعلى بشكل ملحوظ لداء المثقبيات الأفريقي الحيواني قبل وبعد التدخل على حد سواء. وانخفض بشكل ملحوظ انتشار داء المثقبيات الأفريقي الحيواني AAT للمثقبية النشيطة من 5.90٪ إلى 0.05٪ بعد التدخل بينما ارتفعت المثقبية الكونغولية من 8.00٪ إلى 12.20٪.

الاستنتاجات: لقد أدى التدخل إلى انخفاض كبير في انتشار عدوى المثقبية البروسية .s.l، والسل الروديسي المعدي للإنسان والمثقبية النشيطة في قطعان ماشية القرى. وانخفضت نسبة المثقبية البروسية .s.l التي كانت معدية للإنسان، من 1:12 عدوى مثقبية بروسية .s.l قبل التدخل إلى 1:33 بعد التدخل. فمن الواضح أنه من الصعب القضاء على السل الروديسي من الماشية في القرى التي تعرضت لعدوى بشرية. وتعتبر الأدلة على مستويات مرتفعة من داء المثقبيات الأفريقي الحيواني في مجال الثروة الحيوانية ضمن قطعان القرى مؤشراً مفيداً لخطر المثقبية البروسية الروديسية الحادة rHAT في أوغندا. والمراقبة البيطرية والطبية المتكاملة هي المفتاح لنجاح التحكم في أمراض المثقبية البروسية الروديسية الحادة حيوانية المنشأ rHAT.

Translated from English version into Arabic by Free bird, through

TRANSLATORS
WITHOUT BORDERS

在乌干达新发布氏锥虫罗得西亚种（Trypanosoma brucei rhodesiense）感染地区进行以家畜为目标防治人和动物锥虫病效果的村级评估

Louise Hamill, Kim Picozzi, Jenna Fyfe, Beatrice von Wissmann, Sally Wastling, Nicola Wardrop, Richard Selby, Christine Amongi Acup, Kevin L Bardosh, Dennis Muhanguzi, John D Kabasa, Charles Waiswa and Susan C Welburn

摘要

引言：乌干达遭受了一系列非洲锥虫病(HAT)流行的危害，该病亦称昏睡病，是由采采蝇传播的一种疾病。由于进口的感染性牛输入到以往没有锥虫病的区域，因此，受到急性感染布氏锥虫罗得西亚种（Trypanosoma brucei rhodesiense，rHAT)威胁的地区正在扩大。这些地区同时也受到非洲动物锥虫病(AAT)的影响，因此需要综合性疾病控制。

方法：2008 年，政府和社会资本合作(Public Private Partnership)——杜绝昏睡病(SOS)组织在 Dokolo 和 Kaberamaido 地区的 29 个教区对 31 486 头牛进行锥虫病治疗。本研究探讨了干预措施对村级牛群[经历(HAT+ve)或未经历(HAT-ve)近期 rHAT] rHAT 和 AAT 流行的作用。牛群从 20 个村庄抽样，对干预前和干预后 6 个月的牛进行 PCR 筛查，是否感染布氏锥虫（Trypanosoma brucei s.l.）、人感染布氏锥虫罗得西亚种、活动锥虫（Trypanosoma vivax）、刚果锥虫（Trypanosoma congolense savannah）。
结果：干预后，20 个村庄布氏锥虫和人感染布氏锥虫罗得西亚种的流行均显著降低。布氏锥虫罗得西亚种的流行率从 2.40%降至 0.74% ($P < 0.0001$)，并且干预措施在大多数 HAT-ve 村效果显著。牛群感染人锥虫病的村庄数量从 15 个降至 8 个，且布氏锥虫罗得西亚种仅在 HAT+ve 村的牛群中持续存在。感染人的布氏锥虫罗得西亚种的感染率从干预前的 8.3%(95% *CI*=11.1%–5.9%) 降至干预后的 4.1% (95%*CI*=6.8%–2.3%)。最近有人感染病例的村庄(HAT+ve 村)干预前后 AAT 感染水平均较高。活动锥虫的流行率从干预前的 5.90%降至干预后的 0.05%，而刚果锥虫的流行率却从 8.00%增至 12.20%。
结论：干预措施导致布氏锥虫的流行率明显降低，包括人感染布氏锥虫罗得西亚种和牛感染的活动锥虫。感染人的布氏锥虫罗得西亚种的比例从干预前的 1:12 降至干预后的 1:33。与人相比，很显然动物体更难消除布氏锥虫罗得西亚种。在乌干达村级牲畜 AAT 感染水平的升高是 rHAT 风险的有用指标。兽医和医疗监测联合是成功控制人兽共患 rHAT 的关键。

Translated from English version into Chinese by Xin-Yu Feng, edited by Pin Yang, through

Évaluation de l’impact du ciblage du bétail dans la prévention de la trypanosomiase humaine et animale à l’échelle d’un village dans des districts récemment affectés par le parasite *T. b. rhodesiense* en Ouganda

Louise Hamill, Kim Picozzi, Jenna Fyfe, Beatrice von Wissmann, Sally Wastling, Nicola Wardrop, Richard Selby, Christine Amongi Acup, Kevin L Bardosh, Dennis Muhanguzi, John D Kabasa, Charles Waiswa et Susan C Welburn

Résumé
Contexte : l’Ouganda a été touché par une série d’épidémies de trypanosomiase humaine africaine (THA), une maladie transmise par la mouche tsé-tsé aussi appelée la maladie du sommeil. La région affectée par une THA aiguë provoquée par le parasite *Trypanosoma brucei rhodesiense* (THAr) s’étend, stimulée par l’importation de bétail infecté dans des régions auparavant exemptes de la maladie. Ces régions sont aussi affectées par la trypanosomiase animale africaine (TAA) exigeant une lutte intégrée contre la maladie.
Méthodologie : en 2008, le partenariat public-privé, Stamp Out Sleeping Sickness (SOS), a permis le traitement de 31 486 bovins par des trypanocides au sein de 29 paroisses des districts de Dokolo et de Kaberamaido. La présente étude examine l’impact de cette intervention sur la prévalence de la THAr et de la TAA dans le bétail de villages ayant récemment présenté (positifs à la THA) ou non (négatifs à la THA) un cas de THAr. Les troupeaux de bovins de vingt villages ont été échantillonnés et ont subi un dépistage par PCR, avant l’intervention et 6 mois après cette dernière, afin de déterminer la présence ou l’absence des sous-groupes suivants : *Trypanosoma brucei* s.l. ; *T. b. rhodesiense* infectieux pour l’homme ; *Trypanosoma vivax* et *Trypanosoma congolense* type savane.
Résultats : suite à l’intervention, nous avons constaté une diminution significative de la prévalence du parasite *T. brucei* s.l. et de la sous-espèce *T. b. rhodesiense* infectieuse pour l’homme dans le bétail de l’ensemble des 20 villages. La prévalence du parasite *T. b. rhodesiense* a diminué de 2,40 % à 0,74 % ($P < 0,0001$), l’intervention affichant un plus grand impact dans les villages positifs à la THA. Le nombre de villages contenant des bovins hôtes de parasites infectieux pour l’homme a diminué de 15/20 à 8/20, l’infection par le parasite *T. b. rhodesiense* persistant principalement dans

les bovins regroupés dans les villages positifs à la THA (6/8). La proportion des infections par le parasite *T. brucei* s.l. ayant été déterminées de manière définitive comme étant dues au parasite *T. b. rhodesiense* infectieux pour l'homme a diminué suite à l'intervention de 8,3 % (*IC* à 95 % = 11,1 % – 5,9 %) à 4,1 % (*IC* à 95 % = 6,8 % – 2,3 %). Les villages ayant récemment présenté un cas humain (villages positifs à la THA) affichaient une prévalence nettement plus élevée de TAA à la fois avant et après l'intervention. En ce qui concerne la TAA, la prévalence du parasite *T. vivax* a diminué de manière significative de 5,90 % à 0,05 % suite à l'intervention, tandis que celle du parasite *T. congolense* a augmenté, passant de 8,00 % à 12,20 %.
Conclusions : l'intervention a généré une diminution significative de la prévalence des infections par les parasites *T. brucei* s.l., *T. b. rhodesiense* infectieux pour l'homme et *T. vivax dans* les troupeaux de bovins au sein des villages. La proportion des parasites *T. brucei* s.l. Infectieux pour l'homme a diminué de 1:12 infections par les parasites *T. brucei* s.l. avant l'intervention à une proportion de 1:33 suite à l'intervention. Il est clairement plus difficile d'éliminer le parasite *T. b. rhodesiense* dans le bétail de villages ayant été touchés par une trypanosomiase humaine. L'existence démontrée de taux élevés de TAA dans le bétail des troupeaux de village constitue un indicateur utile du risque de THAr en Ouganda. Une surveillance vétérinaire et médicale intégrée est primordiale à la réussite de la lutte contre la THAr.

Translated from English version into French by eric ragu, through

TRANSLATORS
WITHOUT BORDERS

Оценка воздействия на домашний скот мер предотвращения заболевания трипаносомозом людьми и животными на уровне деревень в районах Уганды, недавно поражённых *T. b. rhodesiense*

Луиза Хамилл (Louise Hamill), Ким Пикоцци (Kim Picozzi), Дженна Файф (Jenna Fyfe), Беатрис фон Виссманн (Beatrice von Wissmann), Салли Вэйстлинг (Sally Wastling), Никола Вордроп (Nicola Wardrop), Ричард Селби (Richard Selby), Кристина Амонджи Акуп (Christine Amongi Acup), Кевин Л. Бардош (Kevin L Bardosh), Деннис Мухангузи (Dennis Muhanguzi), Джон Д. Кабаса (John D Kabasa), Чарльз Вайсва (Charles Waiswa) и Сьюзан Велбёрн (Susan C Welburn)

Аннотация
Краткое описание. Уганда пострадала от серии эпидемий человеческого африканского трипаносомоза (HAT) — заболевания, которое переносится мухой цеце и которое также известно как сонная болезнь. Регион поражения острой формой *Trypanosoma brucei rhodesiense* (rHAT) расширяется вследствие импорта инфицированного домашнего скота в районы, где это заболевание ранее не встречалось. В этих районах также распространён животный африканский трипаносомоз (AAT). Это значит, что необходим интегрированный подход к борьбе с болезнями.
Методы. В 2008 году государственно-частное партнёрство Stamp Out Sleeping Sickness (SOS) обработало трипаноцидами 31486 голов домашнего скота в 29 приходах в округах Доколо (Dokolo) и Каберамаидо (Kaberamaido). Данное исследование изучает воздействие этих мер на заражение трипаносомами rHAT и AAT домашнего скота в деревнях, в которых имелись (HAT^{+ve}) или не имелись (HAT^{-ve}) недавние случаи заболевания rHAT. У домашнего скота в двадцати деревнях были взяты пробы, которые были проверены методом ПЦР-диагностики перед обработкой и 6 месяцев после неё на наличие или отсутствие паразитов *Trypanosoma brucei s.l.*, заразного для людей *T. b. Rhodesiense*, *Trypanosoma vivax* и *Trypanosoma congolense savannah*.
Результаты. Во всех 20 деревнях после обработки значительно снизилось распространение *T. brucei* s.l. и заразных для людей подвидов *T. b. rhodesiense*. Заражение *T. b. rhodesiense*

уменьшилось с 2,40% до 0,74% (P < 0,0001), при этом обработка была более эффективной в деревнях HAT^{-ve}. Число деревень, где был домашний скот, заражённый опасными для людей паразитами, уменьшилось с 15/20 до 8/20. Инфицирование *T. b. rhodesiense* в основном преобладало в домашнем скоте в деревнях HAT^{+ve} (6/8). Процент случаев инфицирования *T. brucei s.l.*, которые были несомненно опасны для людей *T. b. rhodesiense,* после обработки снизился с 8,3% (95%CI = 11,1% – 5,9%) до 4,1% (95%CI = 6,8% – 2,3%). Деревни, в которых был недавний случай заболевания человеком (деревни HAT^{+ve}), показали заметно более высокий уровень инфицирования AAT как до обработки, так и после неё. Для AAT заболеваемость *T. vivax* существенно понизилась с 5,90% до 0,05% после обработки, тогда как заболеваемость *T. congolense* выросла с 8,00% до 12,20%.
Выводы. Обработка привела к значительному снижению заражения домашним скотом *T. brucei s.l.*, опасным для людей паразитом *T. b. rhodesiense* и инфекцией *T. vivax*. Процент случаев инфицирования опасным для людей *T. brucei s.l.* уменьшился с 1:12 *T. brucei* s.l. перед обработкой до 1:33 после неё. Очевидно, что гораздо сложнее ликвидировать заражение домашнего скота паразитом *T. b. rhodesiense* в деревнях, где были случаи заболевания людей. Подтверждение повышенных уровней AAT у домашнего скота является важным индикатором риска заражения rHAT в Уганде. Интегрированное ветеринарное и медицинское наблюдение послужит главным фактором успешной борьбы с зоонозной инфекцией rHAT.

Translated from English version into Russian by Natalia Potashnik, through

TRANSLATORS
WITHOUT BORDERS

Evaluación del impacto del ganado meta para la prevención de tripanosomiasis animal y humano a nivel población, en distritos recientemente afectados con *T. b. rhodesiense* en Uganda.

Louise Hamill, Kim Picozzi, Jenna Fyfe, Beatrice von Wissmann, Sally Wastling, Nicola Wardrop, Richard Selby, Christine Amongi Acup, Kevin L Bardosh, Dennis Muhanguzi, John D Kabasa, Charles Waiswa y Susan C Welburn

Resúmen.

Antecedentes: Uganda ha padecido una serie de epidemias de Tripanosomiasis Africana Humana (HAT por su sigla en inglés), una enfermedad transmitida por la mosca tsetse, también conocida como enfermedad del sueño. El área afectada por *Trypanosoma brucei rhodesiense* HAT (rHAT) aguda se está expandiendo, motivado por la importación de ganado infestado en regiones previamente libres de la enfermedad. Estas regiones también están afectadas por Tripanosomiasis Animal Africana (AAT por su sigla en inglés) que demanda un control de la enfermedad integrado.
Metodología: En 2008, la Public Private Partnership, Stamp Out Sleeping Sickness (SOS) trataron con tripanocides 31 486 reses en 29 municipios en los distritos de Dokolo y Kaberamaido.
Este estudio examina el impacto de esta intervención sobre la preponderancia de tripanosomas rHAT y AAT en reses provenientes de poblados que habían experimentado (HAT^{+ve}) o no habían (HAT^{-ve}) experimentado un caso reciente de rHAT. Se muestrearon y controlaron manadas de reses de 20 poblados por parte de PCR, pre-intervención y post-intervención durante 6 meses por presencia o ausencia de *Trypanosoma brucei* s.l.; infeccioso humano *T. b. rhodesiense*; *Trypanosoma vivax* y la savannah *Trypanosoma congolense.*
Resultados: Post-intervención, hubo una disminución significativa en la preponderancia de *T. brucei* s.l. y las sub-especies a nivel infeccioso humano *T. b. rhodesiense* en poblados con ganado bovino en 20 poblaciones. El predominio de *T. b. rhodesiense* se redujo de 2.40% a 0.74% (P < 0.0001), con la intervención que muestra un impacto mayor en poblaciones HAT$^{-ve.}$. La cantidad de poblaciones que contenía ganado bovino refugiando parásitos infecciosos humanos disminuyó de 15/20 a 8/20 con la infección de *T. b. rhodesiense* persistiendo principalmente dentro del ganado bovino en poblaciones HAT^{+ve} (6/8). La proporción de infecciones *T. brucei* s.l. que fue definitivamente infección humana *T. b. rhodesiense* disminuyó después de la intervención de 8.3%

(95%*CI* = 11.1% – 5.9%) to 4.1% (95%*CI* = 6.8% – 2.3%). Las poblaciones que habían experimentado un caso humano reciente (poblaciones HAT^{+ve}) mostraron un predominio significativamente mayor tanto para la pre como para la post-intervención de AAT. En el caso de AAT el predominio de *T. vivax* se redujo significativamente desde 5.90% a 0.05% post-intervención mientras que *T. congolense* aumentó de 8.00% a 12.20%.
Conclusiones: La intervención dio por resultado una disminución significativa en el predominio de *T. brucei* s.l., en el campo infeccioso humano por *T. b. rhodesiense* y en la infección *T. vivax* en manadas de ganado bovino en poblaciones. La proporción de *T. brucei* s.l. que era infeccioso humano disminuyó de infecciones 1:12 *T. brucei* s.l. antes de la intervención a 1:33 post-intervención. Es claramente más difícil eliminar *T. b. rhodesiense* del ganado en poblaciones que han afectado un humano. Prueba de niveles elevados de AAT en ganado dentro de manadas en poblaciones es un indicador de riesgo útil para rHAT en Uganda. Un informe veterinario y médico integrado es clave para tener un control exitoso de rHAT zoonótico.

Translated from English version into Spanish by Ana Tranorte, through

TRANSLATORS
WITHOUT BORDERS
